# Comparative plastome analyses and phylogenetic insights of *Stellaria* (Caryophyllaceae)

**DOI:** 10.3389/fpls.2026.1902479

**Published:** 2026-07-22

**Authors:** Wenqiao Wang, Mujie Shen, Zhiwei Su, Liying Yu, Zhonghui Ma

**Affiliations:** 1 Guangxi Traditional Chinese Medicine (TCM) Resources General Survey and Data Collection Key Laboratory/The Center for Phylogeny and Evolution of Medicinal Plants, National Center for Traditional Chinese Medicine (TCM) Inheritance and Innovation, Guangxi Botanical Garden of Medicinal Plants, Nanning, China; 2 National Engineering Research Center for Southwest Endangered Medicinal Materials Resources Development, Guangxi Botanical Garden of Medicinal Plants, Nanning, China; 3 College of Agriculture, Guangxi University, Nanning, China

**Keywords:** Caryophyllaceae, comparative analysis, infrageneric relationships, plastid genomes, *Stellaria*

## Abstract

*Stellaria* (Caryophyllaceae) comprises approximately 112 species globally, with China serving as a significant center of diversity hosting about 64 species. Despite its taxonomic importance, the genus remains insufficiently studied in China. Previous phylogenetic studies relying on limited DNA barcodes produced weakly supported inferences, while those based on chloroplast genomes are currently lacking. Here, we characterize the chloroplast genome structure and reconstruct its highly resolved infrageneric phylogeny using 60 newly sequenced plastomes. All plastomes displayed a conserved quadripartite structure, with lengths varying from 147,205 bp to 149,409 bp, GC contents ranging from 36.6% to 36.7%, and gene counts spanning 128 to 129 genes. Codon usage patterns were highly conserved with leucine encoded by UUA exhibiting the highest relative synonymous codon usage. A total of 55–74 simple sequence repeats and 42–64 long repeats were detected. Three hypervariable regions—*petN-psbM*, *trnP-rpl33*, and *ycf1* were identified as promising candidate DNA barcodes. Phylogenomic analysis resolved *Stellaria* into three strongly supported major clades and 15 well-defined subclades. The results are largely consistent with the established phylogeny of *Stellaria*. However, we observed certain discrepancies within specific clades. We propose some suggestions for these clades and species based on morphological and molecular evidence. This study provides the first comprehensive phylogenetic framework for *Stellaria* based on the chloroplast genome, establishing a robust foundation for future taxonomic revisions and evolutionary studies.

## 1 Introduction

*Stellaria* L. is one of the largest genera in Caryophyllaceae, and encompasses approximately 112 species worldwide, which are widely distributed from temperate to frigid zones, and some have a circumpolar distribution (Chen and Rabeler, 2001; Sharples and Tripp, 2019). There are about 64 species recorded in China, as a significant distribution center for the genus (Chen and Rabeler, 2001). The genus is distinguished from related genera by its petals deeply bifid to the base and its numerous seeds. It includes common field weeds such as *S. media* (L.) Vill, *S. aquatica* (L.) Scop., and *S. pallida* (Dumort.) Crép.

The infrageneric classification of *Stellaria* has undergone a complex history. Endlicher provided the first systematic treatment, dividing the genus into four sections (Endlicher, 1840). This was followed by significant revisions from Fenzl who introduced a subgeneric rank (Fenzl, 1842), and Turczaninow who proposed six sections (Turczaninow, 1842). The mid-19th to early 20th centuries saw contributions to the ongoing refinement of *Stellaria*’s classification from various botanists. Notably, Schischkin introduced an influential system of six sections, which was widely adopted for decades (Schischkin, 1936). In China, the comprehensive treatment in Flora Reipublicae Popularis Sinicae proposed detailed sectional classifications for regional floras (Ke, 1985; Wu, 1991; Wu and Ke, 1996). However, a major shift occurred with the molecular phylogenetic study conducted by Sharples & Tripp. Using RAD-seq data, they redefined the genus’s boundaries, proposed two new segregate genera, and identified five major evolutionary clades within the core *Stellaria* (Sharples and Tripp, 2019). Their work starkly contrasts with previous morphology-based systems. Consequently, the infrageneric relationships within *Stellaria* remain problematic and unresolved. In particular, certain varieties, such as *Stellaria graminea* var. *viridescens* Maxim., *S. chinensis* var. *ciliata* C. S. Zhu & H. M. Li, and *S. alsine* var. *alpina* (Schur) Hand.-Mazz., render the infrageneric delimitation ambiguous, highlighting the need for a unified taxonomic framework that integrates both morphological and molecular evidence.

Phylogenetic studies are important to reveal relationships between genus and species. Recent phylogenetic studies have reclassified the traditional Caryophyllaceae into 11 tribes (Harbaugh et al., 2010; Greenberg and Donoghue, 2011). These studies have revealed that *Stellaria* constitutes a paraphyletic group, with some species of *Stellaria* being closely related to other genera (Fior et al., 2006; Harbaugh et al., 2010; Greenberg and Donoghue, 2011). Recent studies on tribes Alsineae have further demonstrated that some *Stellaria* species are nested within other genera, including *Arenaria* L., *Cerastium* L., *Minuartia* Loefl., and *Pseudostellaria* Pax (Fior et al., 2006; Harbaugh et al., 2010; Dillenberger and Kadereit, 2014; Sadeghian et al., 2015; Zhang et al., 2017). Sharples and Tripp conducted extensive sampling of *Stellaria* species, clarifying the demarcation between core *Stellaria* and false *Stellaria*. They subsequently divided core *Stellaria* into five clades: Insignes, Larbreae, Nitentes, Plettkeae, and Petiolares, with Larbreae encompassing the majority of species and further subdivided into 13 clades (Sharples, 2019; Sharples and Tripp, 2019). B. Montesinos-Tubée et al. proposed that some species of *Arenaria* and *Pycnophyllopsis* Skottsb. from South America should be reclassified under the genus *Stellaria*, and created a new section: sect. *Plettkea* (Mattf.) Montesinos & Borsch (Montesinos-Tubée and Borsch, 2023). Despite the establishment of the core framework for *Stellaria*, a significant portion of Chinese *Stellaria* species remains under-sampled. Consequently, a comprehensive phylogenetic study of *Stellaria*, particularly focusing on Chinese species, is still warranted.

The infrageneric phylogenetic relationships within the genus *Stellaria*, as inferred from several DNA barcodes, have demonstrated limited support (Yao et al., 2021; Arabi et al., 2022; Xue et al., 2023). The chloroplast genome of angiosperms is characterized by its structural conservation, functional stability, and slow evolutionary rates. With a moderate genome size rich in genetic information, it demonstrates low recombination rates and exists in cells as numerous copies, retaining extensive historical evolutionary genetic information (Badenes and Parfitt, 1995; Nock et al., 2011). Owing to their ability to provide robust evidence for resolving species relationships, chloroplast genomes have been extensively employed in plant phylogenetic studies (Chen et al., 2024; Liu et al., 2024; Xu et al., 2024; Rather et al., 2025). Current phylogenetic frameworks within the genus *Stellaria* worldwide predominantly rely on nuclear gene data, while chloroplast genome research in this genus has received relatively scant attention (Sharples and Tripp, 2019). Frequent nuclear-cytoplasmic conflicts observed in phylogenetic reconstructions raise critical questions regarding whether chloroplast-derived phylogenetic trees could offer more informative insights into intrageneric relationships (Duan et al., 2023; Ren et al., 2024; Tayebi et al., 2024; Thureborn et al., 2024). The potential of chloroplast genome data to elucidate these aspects within *Stellaria* remains to be thoroughly explored through comparative analyses with nuclear genomic datasets.

In this study, we conducted extensive sampling of native Chinese *Stellaria* species and constructed infrageneric phylogenetic trees using chloroplast genome data, with the objective of achieving high-resolution phylogenetic reconstruction. Our research aims to elucidate the phylogenetic relationships and species affinities within Chinese *Stellaria*, thereby providing molecular evidence to support taxonomic revisions at the intrageneric level.

## 2 Materials and methods

### 2.1 Taxon sampling

For phylogenetic studies within *Stellaria*, the selection of taxa was primarily based on the infrageneric classification outlined in the Flora of China (Chen and Rabeler, 2001), excluding false *Stellaria*. All study materials (61 accessions, **Supplementary Table 1**), representing 49 species (including varieties), were collected during field survey from 2019 to 2024. Sixty newly obtained sequences were deposited in GenBank with accession numbers ranging from PZ410351 to PZ410410. These samples provided a largely representative coverage of the native members of *Stellaria* in China. The descriptions of plant morphology were primarily derived from the Flora of China (Chen and Rabeler, 2001), protologues, and field investigations (**Supplementary Table 2**).

### 2.2 DNA sequencing and chloroplast genome assembling, annotating

Total genomic DNA was extracted from silica gel-dried leaf tissues following a modified CTAB method (Doyle and Doyle, 1987). All samples were sequenced by BGI Genomics, and raw reads were filtered with SOAPnuke (Chen et al., 2018) to remove low-quality data, generating high-quality clean reads for downstream analyses. Chloroplast genome assembly for *Stellaria* species was performed using GetOrganelle v1.7.6.1 (Jin et al., 2020) with species-specific parameter adjustments, employing *Stellaria aquatica* (GenBank accession number: MZ570968) as the reference genome. Genome annotation was carried out using Geneious Prime 2022.2.2 (Kearse et al., 2012), the Plastid Genome Annotator (Qu et al., 2019), and CPGAVAS2 (Shi et al., 2019), followed by manual curation against the reference genome within the Geneious Prime 2022.2.2 platform. The circular chloroplast genome map was generated using OGDRAW (Greiner et al., 2019).

### 2.3 Comparative analysis of chloroplast structure and DNA barcoding

To investigate the highly variable regions within the chloroplast genomes of *Stellaria*, a comparative analysis was conducted using 61 representative samples. The chloroplast genomes of *Stellaria* were aligned using MAFFT v7.313 (Katoh and Standley, 2013). Visualization was performed with mVISTA (Frazer et al., 2004) with the LAGAN model (Brudno et al., 2003), using *Stellaria decumbens* as the reference. Then the nucleotide diversity (Pi) was determined using DnaSP v6 (Rozas et al., 2017). The sliding window length was set to 600 bp with a step size of 200 bp.

### 2.4 Codon usage bias analysis

Protein-coding genes were extracted from the 61 *Stellaria* samples for codon usage bias analysis. After removing duplicate sequences, sequences shorter than 300 bp, and sequences not starting with the ATG start codon, 51 sequences were obtained. The relative synonymous codon usage (RSCU) values were calculated using CodonW v1.4.2 (Sharp and Li, 1987). Codon usage visualization for every sample was performed using the online server RSCU-Plot (Hejazi et al., 2025).

### 2.5 Simple sequence repeats and long repeats sequence analysis

Simple sequence repeats (SSRs) in the *Stellaria* chloroplast genomes were detected using the online software MISA (Beier et al., 2017). The minimum repeat thresholds were set as follows: 10 repeats for mononucleotide SSRs, 5 for dinucleotide, 4 for trinucleotide, 3 for tetranucleotide, 3 for pentanucleotide, and 3 for hexanucleotide SSRs. Long repeat sequences, including forward, palindromic, reverse, and complementary repeats, were detected using REPuter (Kurtz et al., 2001), with the minimum repeat length set to 30 bp and the Hamming distance set to 3.

### 2.6 Chloroplast phylogenetic analysis

Numerous studies on Caryophyllaceae have clearly demonstrated that *Cerastium* is the sister group of *Stellaria* (Harbaugh et al., 2010; Greenberg and Donoghue, 2011; Sharples and Tripp, 2019; Arabi et al., 2022). *Cerastium glomeratum* Thuill. (GenBank: OP104127) was well assembled and annotated, and here it was designated as the outgroup. Phylogenetic analysis was conducted on PhyloSuite v1.2.2 (Zhang et al., 2020), and whole chloroplast genome sequences were aligned with MAFFT v7.313 (Katoh and Standley, 2013), and manually refined after trimming with trimAI (Capella-Gutierrez et al., 2009). Model selection was conducted using ModelFinder under the AIC criterion (Kalyaanamoorthy et al., 2017). GTR+F+I+G4 was selected to perform following phylogenetic analysis. Maximum likelihood (ML) analysis was performed in IQ-TREE v1.6.8 (Lam-Tung et al., 2015) with default parameters and 1000 bootstrap replicates. Bayesian inference (BI) was implemented in MrBayes 3.2.6 (Ronquist et al., 2012), running Monte Carlo Markov chains (MCMCs) for 2,000,000 generations, sampling every 100 generations. The first 25% of trees were discarded as burn-in, and convergence was confirmed when the average standard deviation of split frequencies fell below 0.01. The candidate DNA barcodes sequences were extracted from whole chloroplast genome using PhyloSuite v1.2.2 (Zhang et al., 2020), aligning with MAFFT v7.313 (Katoh and Standley, 2013), and manually refined after trimming with trimAI (Capella-Gutierrez et al., 2009). Model selection was conducted using PartitionFinder 2 under the AIC criterion (Lanfear et al., 2017). Bayesian inference (BI) is conducted as the above.

## 3 Results

### 3.1 Plastome characteristics

**Figure 1:**
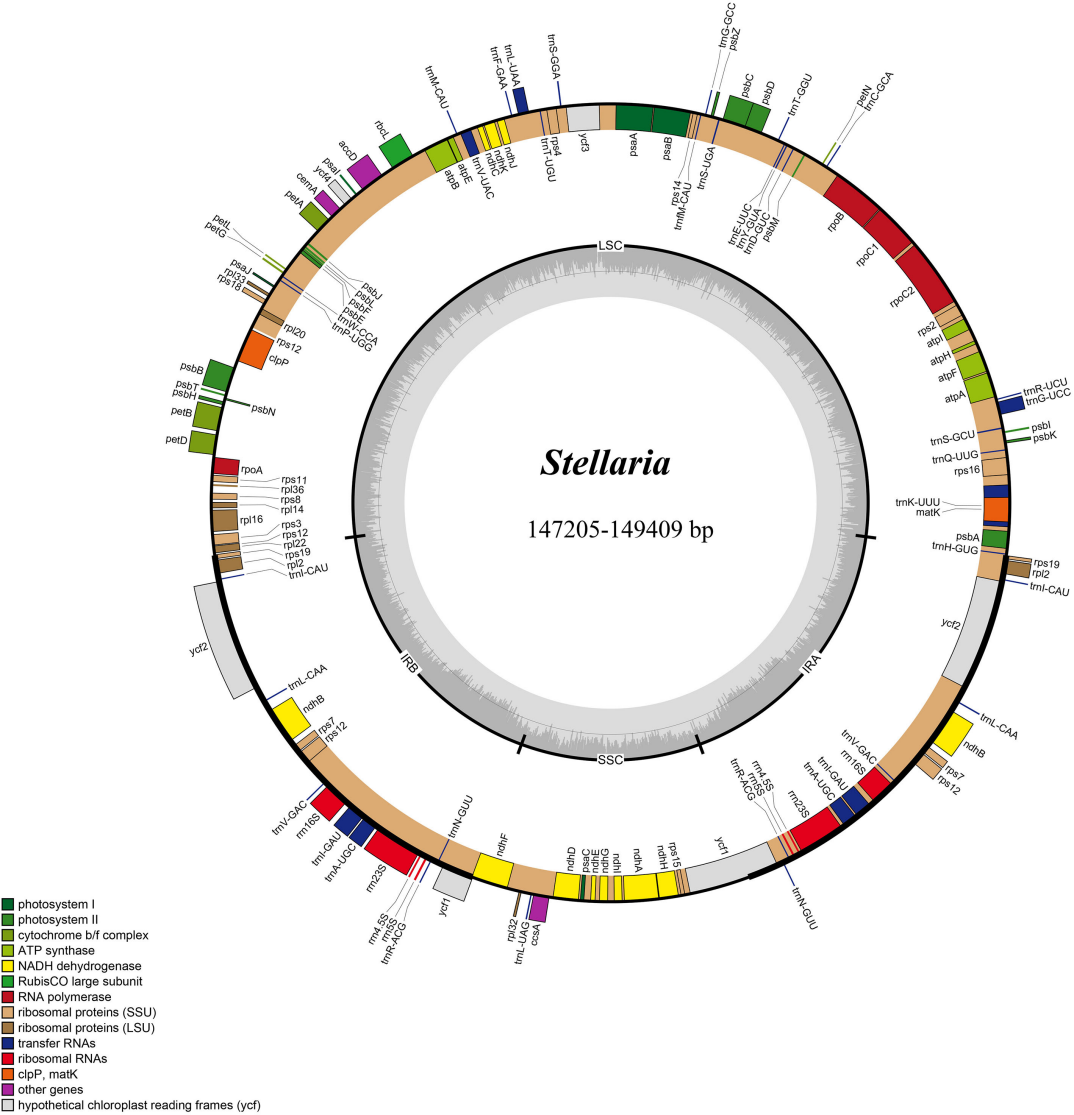
Chloroplast genome map of *Stellaria* species.

The chloroplast genomes of *Stellaria* species exhibited a typical quadripartite structure (the total length of 147,205–149,409 bp), comprising a large single-copy (LSC) region (the length of 79,264–80,941 bp), a small single-copy (SSC) region (the length of 16,599–16,873 bp), and two inverted repeat (IR) regions (the length of 25,488–25,812 bp) (**Figure 1**; **Table 1**). The overall GC content ranged from 36.6% to 36.7%, with specific values of 34.4%–34.5% for the LSC, 29.4%–29.6% for the SSC, and 42.2%–42.5% for the IR regions (**Table 1**). A total of 128–129 genes were annotated, including 83–84 protein-coding genes, 37 tRNA genes, and 8 rRNA genes. Among these, six protein-coding genes (*rps12*, *rps7*, *rpl2*, *ndhB*, *ycf1*, and *ycf2*), seven tRNA genes (*trnA-UGC*, *trnI-CAU*, *trnI-GAU*, *trnL-CAA*, *trnN-GUU*, *trnR-ACG*, and *trnV-GAC*), and all rRNA genes (*rrn16*, *rrn23*, *rrn4.5*, and *rrn5*) possessed two copies (**Table 2**). Ten protein-coding genes (*atpF*, *ndhA*, *ndhB*×2, *petA*, *petB*, *petD*, *rpl16*, *rps16*, and *rpoC1*) and eight tRNA genes (*trnA-UGC*×2, *trnG-UCC*, *trnI-GAU*×2, *trnK-UUU*, *trnL-UAA*, and *trnV-UAC*) contained one intron, while four genes (*rps12*×2, *clpP*, and *ycf3*) contained two introns (**Table 2**). Additionally, *rps19* was a pseudogene, and *rps12* was trans-spliced gene.

**Table 1:** Features of 61 *Stellaria* chloroplast genomes.

Taxon	Genome size (bp)	LSC size (bp)	SSC size (bp)	IR size (bp)	GC content (%)	GC in LSC (%)	GC in SSC (%)	GC in IR (%)	Number of CDS	Number of tRNA	Number of rRNA
*S. abaensis*	148507	80495	16686	25663	36.6	34.5	29.5	42.3	83	37	8
*S. alaschanica*	148403	80364	16713	25663	36.7	34.5	29.5	42.3	83	37	8
*S. alsine*	147877	79788	16735	25677	36.7	34.5	29.4	42.3	83	37	8
*S. alsine* var. *alpina 1*	148631	80592	16699	25670	36.6	34.4	29.5	42.3	83	37	8
*S. alsine* var. *alpina 2*	148631	80592	16699	25670	36.6	34.4	29.5	42.3	83	37	8
*S. amplexicaulis*	148704	80669	16727	25654	36.7	34.5	29.6	42.4	83	37	8
*S. arenarioides*	148596	80593	16647	25678	36.6	34.5	29.6	42.3	83	37	8
*S. brachypetala*	148354	80314	16712	25664	36.7	34.5	29.5	42.3	83	37	8
*S. bungeana* var. *stubendorfii*	148700	80851	16873	25488	36.6	34.5	29.6	42.4	83	37	8
*S. chinensis*	148673	80613	16710	25675	36.7	34.5	29.6	42.4	83	37	8
*S. chinensis* var. *ciliata*	148620	80593	16709	25659	36.6	34.5	29.5	42.4	83	37	8
*S. crassifolia*	148534	80468	16730	25668	36.7	34.5	29.5	42.4	83	37	8
*S. decumbens 1*	148663	80624	16721	25659	36.6	34.5	29.5	42.3	83	37	8
*S. decumbens 2*	148502	80449	16725	25664	36.6	34.5	29.5	42.3	83	37	8
*S. decumbens 3*	148661	80605	16728	25664	36.6	34.4	29.5	42.3	83	37	8
*S. decumbens* var. *arenarioides*	148636	80583	16723	25665	36.6	34.4	29.5	42.3	83	37	8
*S. dianthifolia 1*	148691	80687	16702	25651	36.6	34.5	29.5	42.4	83	37	8
*S. dianthifolia 2*	148683	80679	16702	25651	36.6	34.5	29.5	42.4	83	37	8
*S. dianthifolia 3*	148689	80685	16702	25651	36.6	34.5	29.5	42.4	83	37	8
*S. dianthifolia 4*	148690	80686	16702	25651	36.6	34.5	29.5	42.4	83	37	8
*S. discolor*	148724	80661	16755	25654	36.6	34.4	29.5	42.4	83	37	8
*S. filicaulis*	148752	80641	16761	25675	36.6	34.4	29.5	42.4	83	37	8
*S. graminea* var. *viridescens*	148695	80460	16705	25765	36.6	34.5	29.6	42.3	84	37	8
*S. infracta*	148913	80723	16690	25750	36.7	34.5	29.6	42.5	83	37	8
*S. irrigua 1*	148479	80438	16713	25664	36.7	34.5	29.5	42.3	83	37	8
*S. irrigua 2*	148597	80561	16710	25663	36.6	34.5	29.5	42.3	83	37	8
*S. lanata*	148703	80686	16665	25676	36.6	34.4	29.6	42.4	83	37	8
*S. lanipes*	148733	80677	16726	25665	36.6	34.4	29.5	42.3	83	37	8
*S. longifolia*	148619	80519	16778	25661	36.6	34.5	29.4	42.4	83	37	8
*S. mainlingensis*	148703	80686	16665	25676	36.6	34.5	29.6	42.4	83	37	8
*S. maximowiczii*	148749	80691	16708	25675	36.6	34.4	29.5	42.3	83	37	8
*S. media*	147363	79400	16763	25600	36.7	34.5	29.4	42.3	83	37	8
*S. neglecta*	148422	80569	16873	25490	36.6	34.5	29.6	42.4	83	37	8
*S. nipponica*	148710	80680	16728	25651	36.7	34.5	29.5	42.4	83	37	8
*S. omeiensis*	148597	80659	16602	25668	36.6	34.4	29.6	42.3	83	37	8
*S. oxycoccoides*	148787	80676	16755	25678	36.6	34.4	29.5	42.4	83	37	8
*S. pallida*	147205	79264	16749	25596	36.7	34.5	29.5	42.3	83	37	8
*S. palustris*	148534	80561	16645	25664	36.6	34.5	29.5	42.4	83	37	8
*S. parviumbellata*	148742	80706	16712	25662	36.6	34.5	29.6	42.3	83	37	8
*S. pentastyla 1*	148640	80630	16712	25649	36.6	34.5	29.5	42.4	83	37	8
*S. pentastyla 2*	148641	80629	16712	25650	36.6	34.5	29.5	42.4	83	37	8
*S. pentastyla 3*	148621	80611	16712	25649	36.6	34.5	29.5	42.4	83	37	8
*S. pentastyla 4*	148660	80643	16715	25651	36.6	34.5	29.5	42.4	83	37	8
*S. patens*	148635	80588	16711	25668	36.7	34.5	29.6	42.4	83	37	8
*S. petiolaris*	148647	80682	16663	25651	36.6	34.5	29.5	42.4	83	37	8
*S. pilosoides*	148685	80648	16727	25655	36.6	34.5	29.5	42.3	83	37	8
*S. polyantha*	148584	80515	16719	25675	36.6	34.4	29.5	42.3	83	37	8
*S. pusilla*	148506	80470	16708	25664	36.7	34.5	29.6	42.4	83	37	8
*S. radians*	149409	80941	16844	25812	36.6	34.4	29.5	42.2	84	37	8
*S. salicifolia*	148535	80566	16659	25655	36.6	34.5	29.5	42.4	83	37	8
*S. sikkimensis*	148739	80722	16691	25663	36.6	34.5	29.6	42.4	83	37	8
*S. soongorica*	148687	80602	16745	25670	36.6	34.5	29.5	42.3	83	37	8
*S. souliei*	148692	80688	16702	25651	36.6	34.5	29.5	42.4	83	37	8
*S. longipedicellata*	148649	80648	16601	25700	36.6	34.4	29.6	42.4	83	37	8
*S. uda*	148641	80648	16685	25654	36.6	34.5	29.6	42.3	83	37	8
*S. uda*	148663	80688	16683	25646	36.6	34.5	29.6	42.3	83	37	8
*S.* sp	148658	80687	16663	25654	36.6	34.5	29.5	42.4	83	37	8
*S. vestita*	148666	80622	16706	25669	36.7	34.5	29.6	42.4	83	37	8
*S. winkleri*	148483	80468	16687	25664	36.7	34.5	29.6	42.4	83	37	8
*S. yunnanensis*	148673	80644	16727	25651	36.6	34.5	29.5	42.4	83	37	8

**Table 2:** Genes in the chloroplast genome of *Stellaria* species.

Gene group	Gene name
Subunits of photosystem I	*psaA, psaB, psaC, psaI, psaJ*
Subunits of photosystem II	*psbA, psbB, psbC, psbD, psbE, psbF, psbH, psbI, psbJ, psbK, psbL, psbM, psbN, psbT, psbZ*
Subunits of NADH dehydrogenase	ndhA*, ndhB*×2*, ndhC, ndhD, ndhE, ndhF, ndhG, ndhH, ndhI, ndhJ, ndhK*
Subunits of cytochrome b/f complex	petA, petB*, petD*, petG, petL, petN
Subunits of ATP synthase	atpA, atpB, atpE, atpF*, atpH, atpI
Large subunit of rubisco	*rbcL*
Proteins of large ribosomal subunit	rpl14, rpl16*, rpl2×2, rpl20, rpl22, rpl32, rpl33, rpl36
Proteins of small ribosomal subunit	*rps19*^ψ^, rps11, rps12**×2, rps14, rps15, rps16*, rps18, rps19, rps2, rps3, rps4, rps7×2, rps8
Subunits of RNA polymerase	rpoA, rpoB, rpoC1*, rpoC2
Ribosomal RNAs	*rrn16S*×2*, rrn23S*×2*, rrn4.5S*×2*, rrn5S*×2
Transfer RNAs	trnA-UGC*×2, trnC-GCA, trnD-GUC, trnE-UUC, trnF-GAA, trnG-GCC, trnG-UCC*, trnH-GUG, trnI-CAU×2, trnI-GAU*×2, trnK-UUU*, trnL-CAA×2, trnL-UAA*, trnL-UAG, trnM-CAU, trnN-GUU×2*, trnP-UGG, trnQ-UUG, trnR-ACG*×2*, trnR-UCU, trnS-GCU, trnS-GGA, trnS-UGA, trnT-GGU, trnT-UGU, trnV-GAC*×2, trnV-UAC*, trnW-CCA, trnY-GUA, trnfM-CAU
Maturase	*matK*
Protease	clpP**
Envelope membrane protein	*cemA*
Acetyl-CoA carboxylase	*accD*
c-type cytochrome synthesis gene	*ccsA*
Conserved hypothetical chloroplast ORF	*ycf1*×2*, ycf2*×2, ycf3**, ycf4

×2: Two gene copies in inverted repeats; *: gene containing a single intron; **: gene containing two introns; ψ: pseudogene.

### 3.2 Comparative analysis of chloroplast structure and DNA barcoding

**Figure 2:**
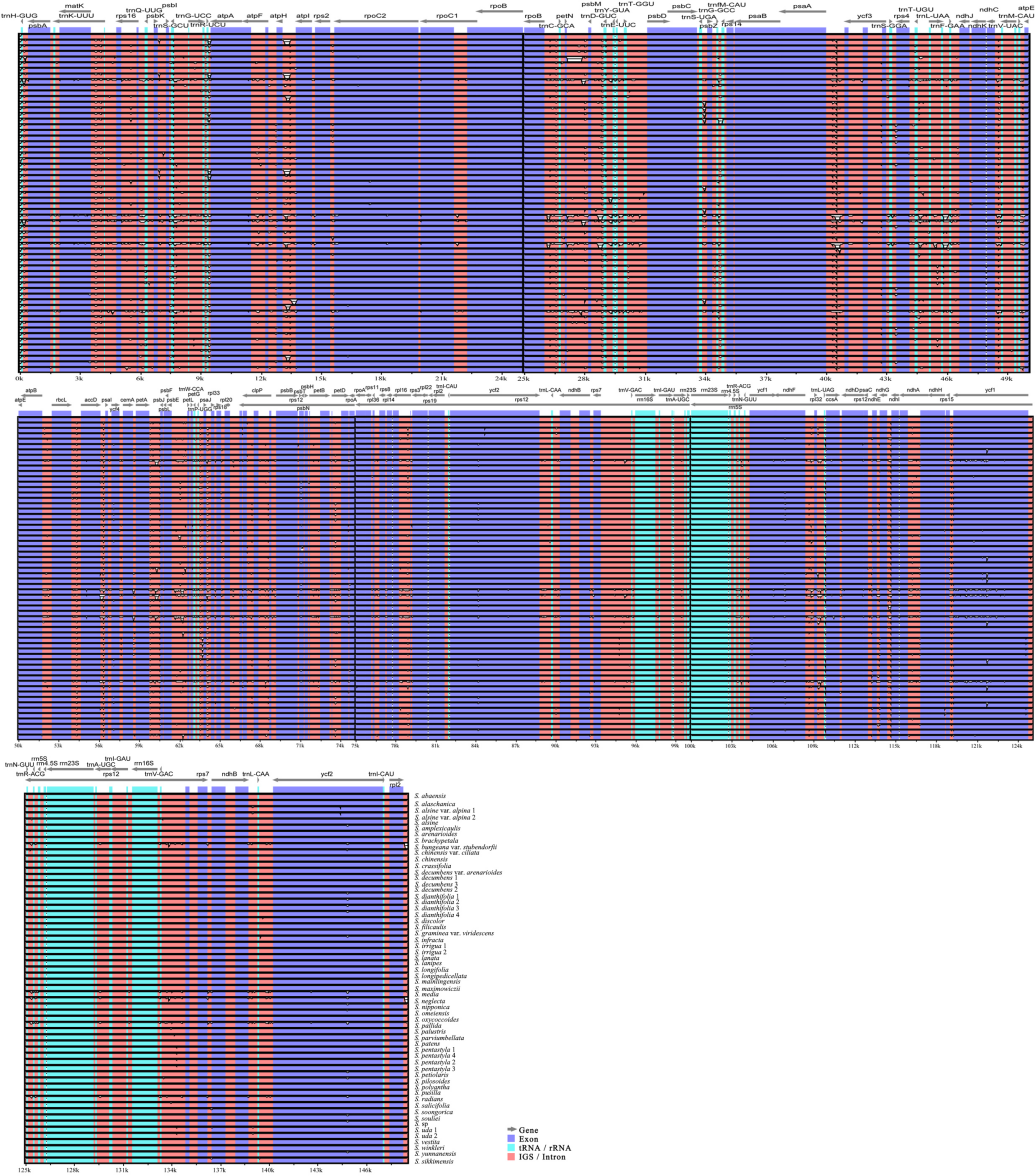
Complete chloroplast genome alignment of *Stellaria* species.

**Figure 3:**
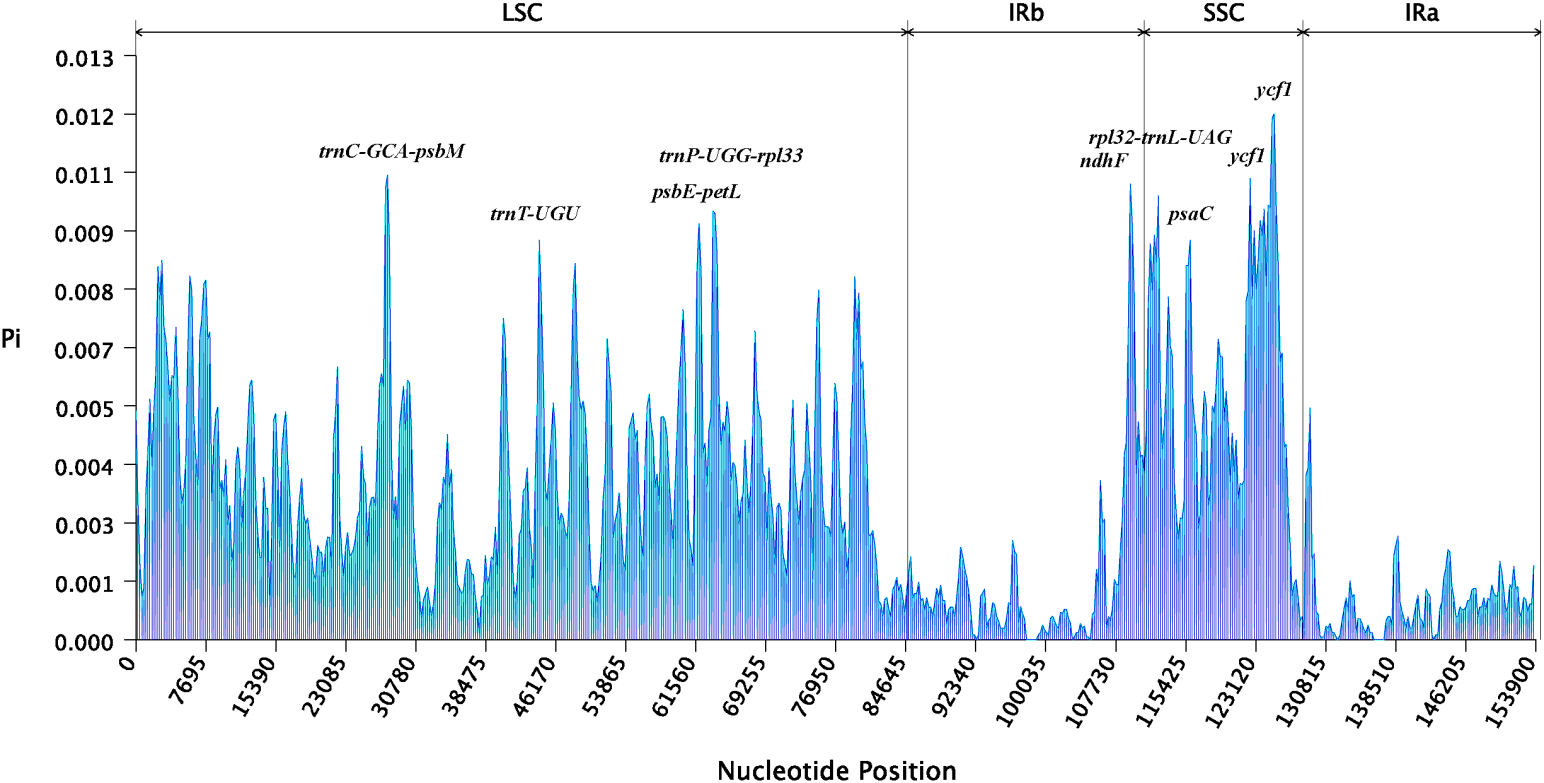
Nucleotide diversity of 61 *Stellaria* species chloroplast genomes.

The chloroplast genome of *Stellaria* investigated in this study exhibits a conserved structure with no observed rearrangements (**Figure 2**). Then the mVISTA results indicated high conservation among the *Stellaria* chloroplast genomes. The following hypervariable regions were identified: *trnH-GUG-psbA*, *trnK-UUU*, *trnG-UCC-atpA*, *atpH-atpI*, *rpoB-trnC-GCA*, *petN-psbM*, *psbM-trnD-GUC*, *psaA-ycf3*, *ndhC-trnV-UAC*, *rbcL-accD*, *accD-psaI*, *petA-psbL*, *trnP-UGG-psaJ-rpl33*, *ndhG-ndhI*, *rps15-ycf1*, and *ycf1* (**Figure 2**). The DnaSP results also confirmed high conservation among the *Stellaria* chloroplast genomes, with the LSC and SSC regions exhibiting higher variation than the IR regions. The highest Pi value was 0.01211, found in the *ycf1* region. Additional hypervariable regions included: *trnC-psbM*, *trnT-UGU*, *psbE-petL*, *trnP-UGG-rpl33*, *ndhF*, *rpl32-trnL-UAG*, and *psaC* (**Figure 3**). Based on these combined results, the following three hypervariable fragments are proposed as candidate DNA barcodes for *Stellaria* studies: *petN-psbM*, *trnP-rpl33*, and *ycf1*. The Bayesian phylogenetic tree based on concatenated DNA barcodes is largely consistent with the chloroplast genome-based phylogeny, with only a few species showing discrepancies (**Supplementary Figure 1**). These DNA barcodes are capable of discriminating the vast majority of species.

### 3.3 Codon usage bias analysis

**Figure 4:**
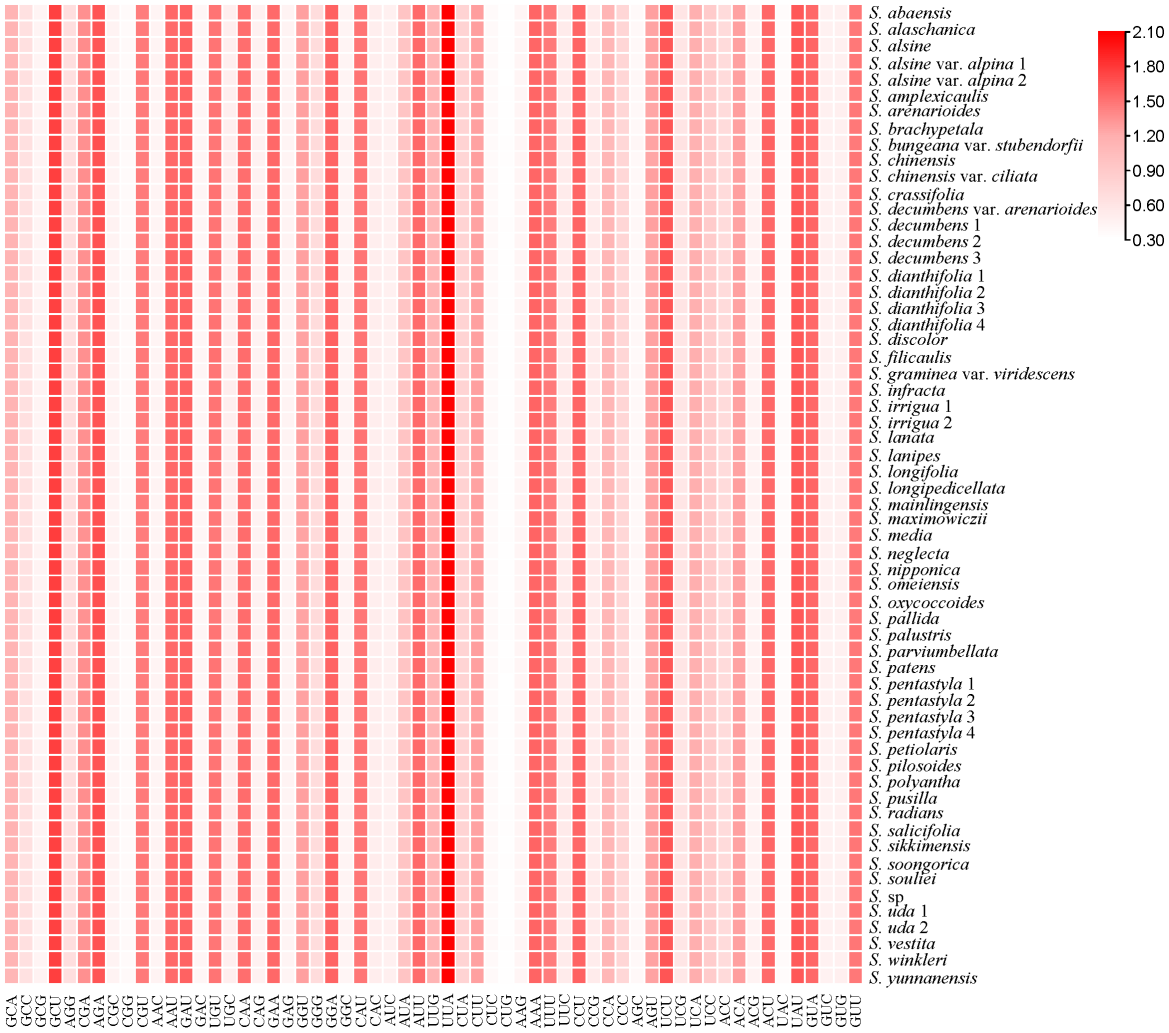
Number of codons and relative synonymous codon usage values of the plastid genome of 61 *Stellaria* species.

The synonymous codon usage patterns among the *Stellaria* species were similar. Leucine (UUA) exhibited the highest RSCU values, ranging from 2.07 to 2.09 (**Figure 4**; **Supplementary Figure 2**; **Supplementary Table 2**). Among the 59 synonymous codons in the *Stellaria* chloroplast genomes, 29 had RSCU values greater than 1, identifying them as high-frequency codons, and 28 of these ended with A/U. Conversely, 30 codons had RSCU values less than 1, identifying them as low-frequency codons, and 28 of these ended with G/C (**Figure 4**; **Supplementary Figure 2**; **Supplementary Table 3**).

### 3.4 Simple sequence repeats and long repeats sequence analysis

**Figure 5:**
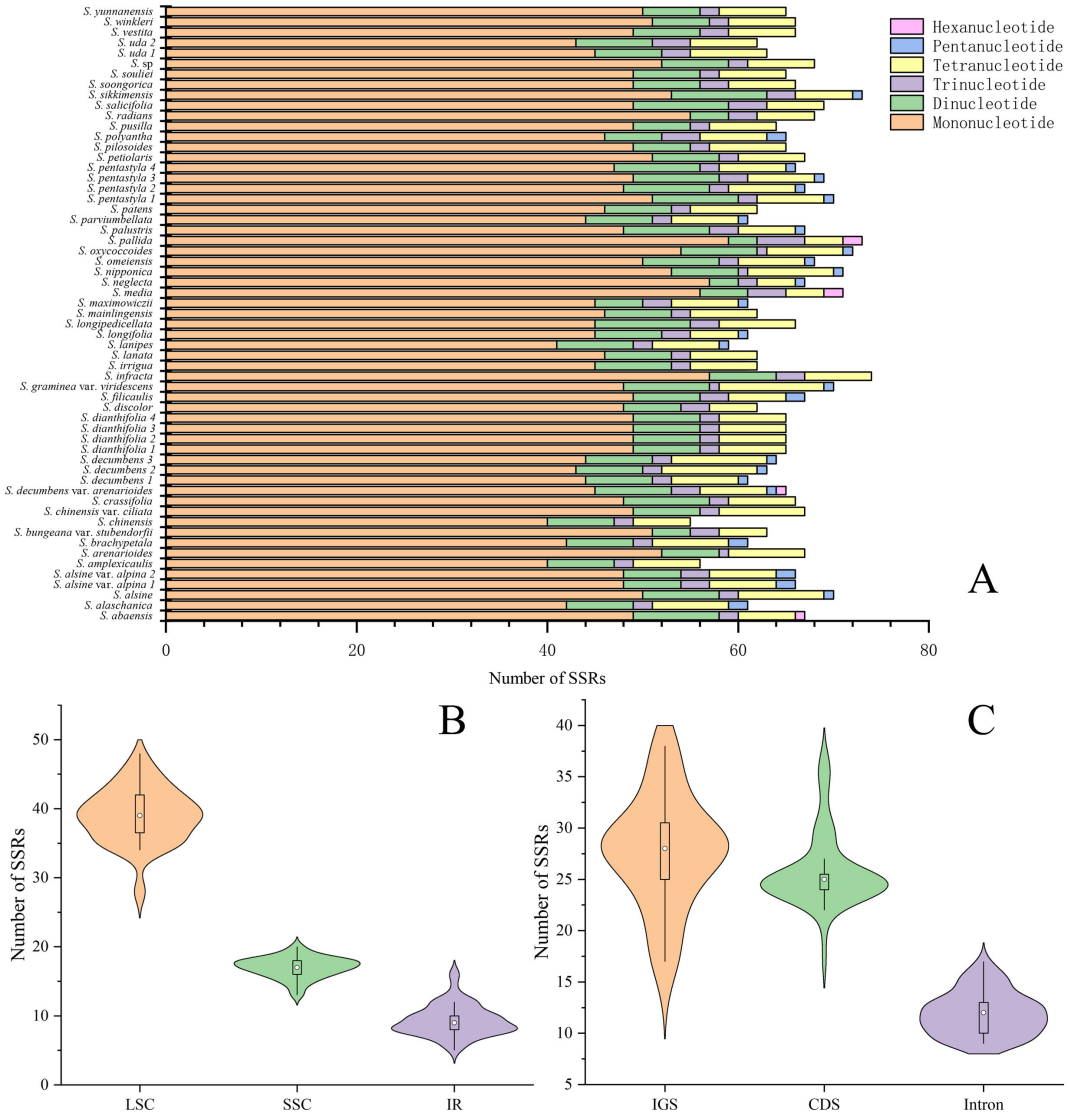
Analysis of simple sequence repeats (SSRs) in 61 Stellaria plastid genomes. **(A)** Number of six SSR repeat types; **(B)** Number of SSRs in the LSC, SSC, and IR regions; **(C)** Number of SSRs in the coding sequences (CDS), intergenic regions (IGS), and introns.

The number of SSRs in the *Stellaria* species ranged from 55 to 74, comprising six types: mononucleotide (the number of 40-59), dinucleotide (the number of 3-10), trinucleotide (the number of 1-5), tetranucleotide (the number of 4-11), pentanucleotide (the number of 0-2), and hexanucleotide (the number of 0-2) repeats. Mononucleotide repeats had the highest proportion (68.18%-85.07%) (**Figure 5**; **Supplementary Table 4**). SSRs were predominantly located in the LSC region (50.00%-67.16%), with fewer found in the SSC (18.57%-32.73%) and IR (7.94%-21.92%) regions. They were more frequent in intergenic regions (25.37%-57.35%) and protein-coding regions (25.00%-55.22%), and less frequent in intronic regions (13.43%-24.29%) (**Figure 5**; **Supplementary Table 3**).

**Figure 6:**
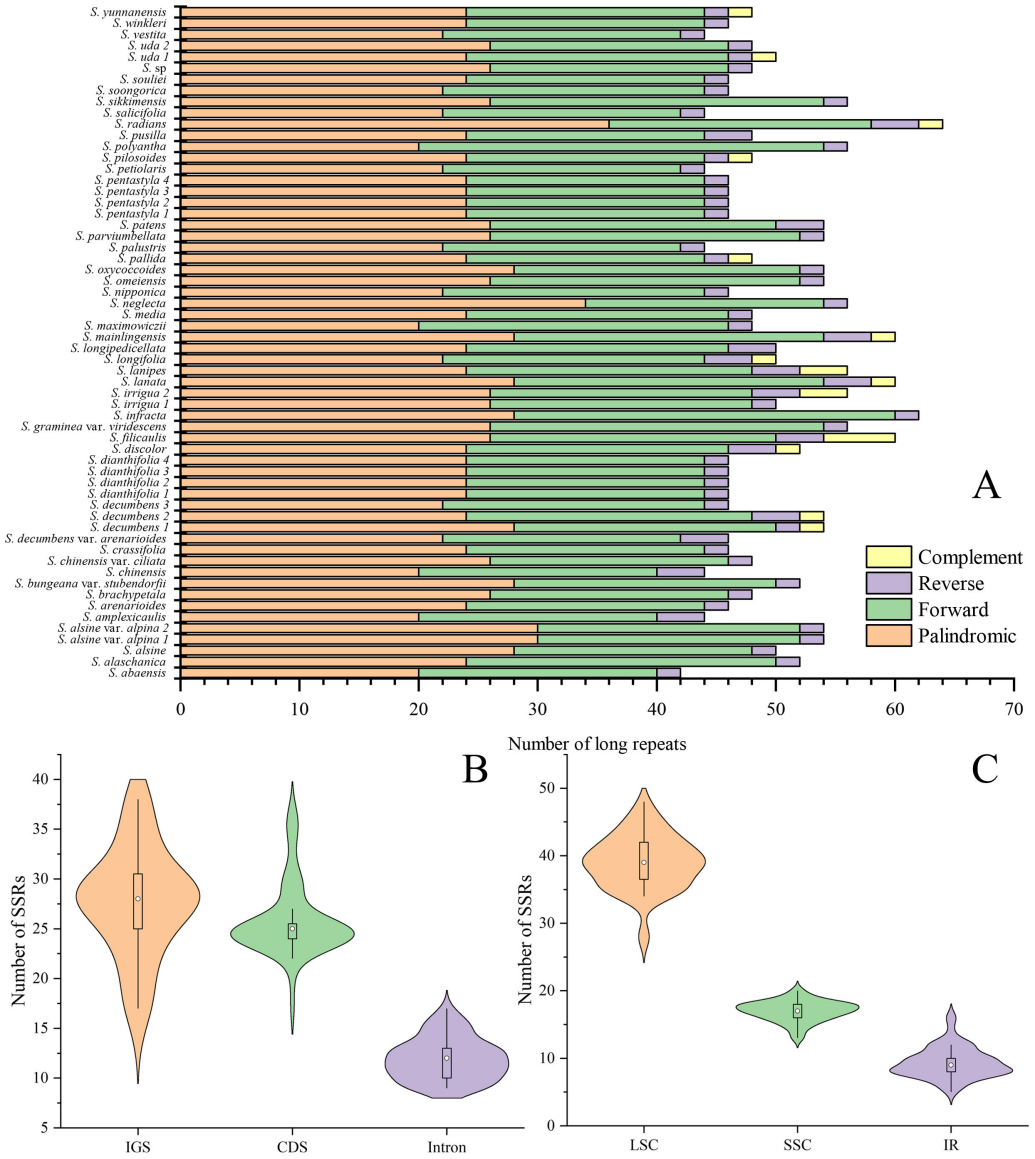
Analysis of long repeats in 61 *Stellaria* plastid genomes. **(A)** Number of forward, palindromic, reverse and complement long repeats; **(B)** Number of long repeats in LSC, SSC, and IR regions; **(C)** Number of long repeats in the coding regions (CDS), intergenic region (IGS), and introns.

The number of long repeat sequences in *Stellaria* ranged from 42 to 64, comprising four types: forward (the number of 20-34), palindromic (the number of 20-36), reverse (the number of 2-4), and complementary (the number of 0-6) repeats. Forward repeats (34.38%-60.71%) and palindromic repeats (35.71%-60.71%) accounted for the highest proportions (**Figure 6**; **Supplementary Table 4**). Long repeats were frequently located in the LSC (50.00%-66.07%) and IR (25.00%-45.16%) regions, with fewer in the SSC region (3.70%-12.50%). They were more abundant in intergenic regions (30.43%-48.39%) and protein-coding regions (30.00%-48.15%), and less abundant in intronic regions (16.67%-35.19%) (**Figure 6**; **Supplementary Table 5**).

### 3.5 Phylogenetic analysis

**Figure 7:**
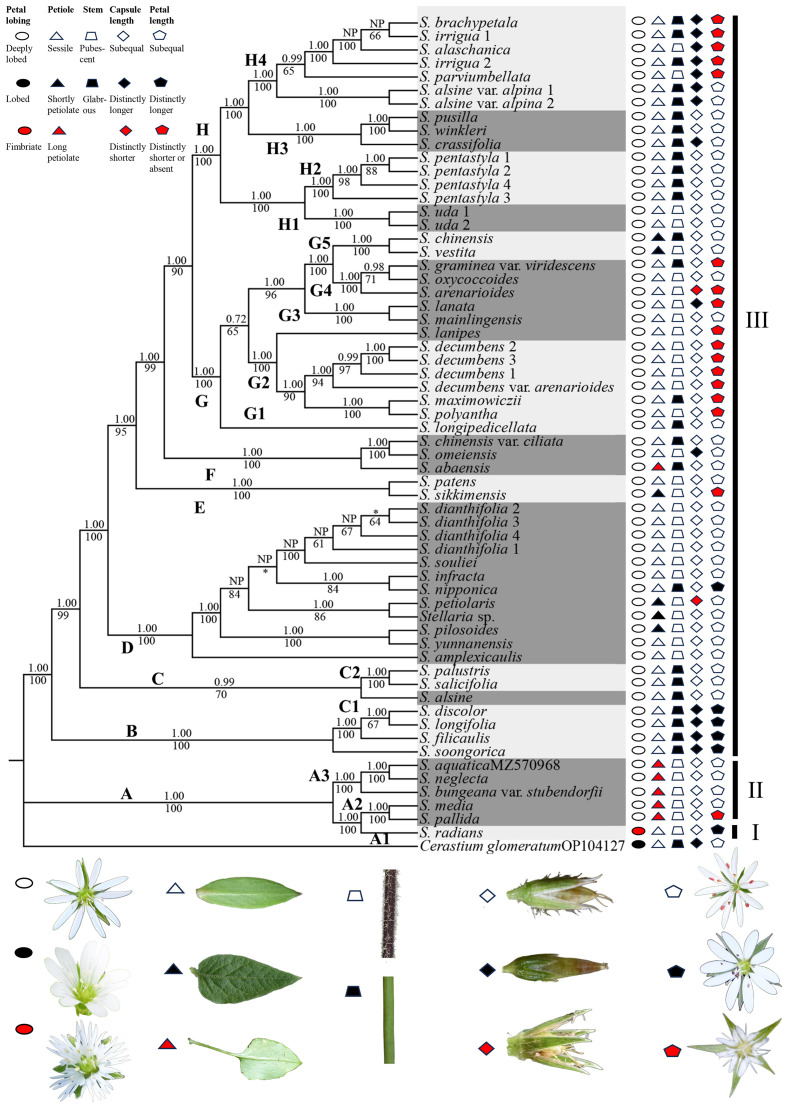
Maximum likelihood (ML) tree of *Stellaria* based on whole chloroplast genomes. Posterior probability (PP) in Bayesian inference (BI) and bootstrap (BS) value in ML analysis are indicated above and below the stem branch of each phylogenetic node, respectively. NP indicates the topology was not present in BI analysis. * indicates that the PP or BS value is less than 0.5 or 50%.

The genus *Stellaria* formed a sister clade to *Cerastium*, and eight major highly supported clades (A–H) were identified within *Stellaria* (**Figure 7**). Clade A consists of three subclades (A1, A2, and A3), with A3 being sister to the clade formed by A1 and A2, which are themselves sister groups (**Figure 7**). Clade B comprises *S. soongorica* Roshev., *S. filicaulis* Makino, *S. longifolia* Muhl. ex Willd., and *S. discolor* Turcz. (**Figure 7**). Clade C includes two sister subclades, C1 and C2. Clade D is composed of species such as *S. petiolaris* Hand.-Mazz., *S. pilosoides* Shi L. Chen, Rabeler & Turland, and *S. dianthifolia* F. N. Williams. Clade E contains *S. patens* D. Don and *S. sikkimensis* Hook.f., while clade F consists of *S. abaensis* H. F. Xu & Z. H. Ma, *S. chinensis* var. *ciliata* C. S. Zhu & H. M. Li, and *S. omeiensis* C. Y. Wu & Tsui ex P. Ke. Clade G has a more complex structure, including subclades G1, G2, G3, G4, and G5, where G1 is sister to the clade containing G2, G3, G4, and G5. G2 is sister to the group formed by G3, G4, and G5. G3 is sister to the clade of G4 and G5, and G4 and G5 are sister to each other (**Figure 7**). Finally, clade H is divided into four subclades (H1, H2, H3, and H4), in which the group (H1 + H2) is sister to (H3 + H4), and within these, H1 and H2 are sister clades, as are H3 and H4 (**Figure 7**).

## 4 Discussion

### 4.1 Comparative genomics

The chloroplast genomes of *Stellaria* species are highly conserved in terms of size (147,205–149,409 bp), GC contents (36.6–36.7%), and gene count (128–129 genes) (**Table 1**). Plastomes within the same species are also highly conserved, exhibiting similar genome size, GC content, SSR counts, and gene order (**Table 1**; **Figures 5**, **6**; **Supplementary Tables 4**, **5**). Notably, the plastome of *S. irrigua* 2 is 118 bp longer than that of *S. irrigua* 1 (148,597 bp vs. 148,479 bp), primarily due to an expanded LSC region (**Table 1**). Additionally, *S. irrigua* 2 contains six more long SSRs than *S. irrigua* 1 (56 vs. 50) (**Figure 6**; **Supplementary Table 5**). However, their GC content and gene order remain largely consistent. The *Cerastium* and *Pseudostellaria* are close to *Stellaria* (Harbaugh et al., 2010; Greenberg and Donoghue, 2011; Sharples and Tripp, 2019; Arabi et al., 2022). Notably, the chloroplast genome lengths of *Cerastium* (147,945–148,523 bp) and *Pseudostellaria* (149,406–149,765 bp) are largely comparable to those of *Stellaria* (Milarska et al., 2023; Zhang et al., 2023). Regional variation in GC content is evident across the *Stellaria* chloroplast genome, which also harbors the *rps12* trans-spliced gene—a feature widely conserved among angiosperms (Kang et al., 2017; Carlos del Valle et al., 2019; Androsiuk et al., 2020; Chen et al., 2022; Kamra et al., 2023).

The number of SSRs in *Stellaria* species ranges from 55 to 74, in marked contrast to the sister genus *Cerastium*, which contains only 16–23 SSRs (Milarska et al., 2023), a substantially lower count. Repeat sequences are ubiquitous in plant chloroplast genomes and have been widely employed in studies of genetic variation, population structure, and species identification (Cavalier-Smith, 2002; Perdereau et al., 2014; Lin et al., 2022).

An RSCU value greater than 1 indicates strong codon usage bias, value equal to 1 denotes no bias, and value less than 1 reflects weak bias (Sharp and Li, 1986). Of the 59 synonymous codons identified in the *Stellaria* chloroplast genome, 29 exhibit RSCU values exceeding 1, indicative of strong codon usage bias, with 28 of these codons terminating in A/U. The remaining 30 codons display RSCU values below 1, indicating weak bias, of which 28 ended in G/C. The Leucine codon (UUA) is the most frequently used in the *Stellaria* chloroplast genome.

### 4.2 DNA barcoding within *Stellaria*

Conventional DNA barcodes, such as ITS, *matK*, *rps16*, *trnL-F*, and *rbcL*, have been widely employed in molecular phylogenetic studies of the tribe Alsineae. However, they frequently demonstrate limited discriminatory power at the intra-generic level within *Stellaria* (Yao et al., 2021; Arabi et al., 2022; Xue et al., 2023). In contrast, the *petN-psbM*, *trnP-rpl33*, and *ycf1* DNA fragments selected in the present study demonstrate high nucleotide diversity within *Stellaria* and show strong potential for species classification and identification at the genus level (**Supplementary Figure 1**). Moreover, accumulating evidence indicates that the complete chloroplast genome, when utilized as a super-barcode, can effectively resolve closely related species (Cai et al., 2021; Ji et al., 2021; Wu et al., 2021; Zhang et al., 2023). Consistent with these findings, the present study demonstrates that chloroplast genomes provide high-resolution discrimination among closely related *Stellaria* species. Given the continuing decline in sequencing costs, whole chloroplast genome sequencing holds considerable promise as a robust tool for future classification and identification within the genus *Stellaria*.

### 4.3 Infrageneric relationships of *Stellaria*

We classified the native Chinese *Stellaria* species into three major clades (I, II, III), which largely correspond to the five clades identified in global *Stellaria* studies (two clades are absent from China). Our results demonstrate that *Stellaria radians* L., which bears fimbriate petals, is nested within clade A, whereas the global study placed *S. radians* together with other non-fimbriate species in a separate clade (Sharples and Tripp, 2019). Historically, both the Flora of the U.S.S.R and the Flora Reipublicae Popularis Sinicae assigned *S. radians* to a distinct section (the sect. *Fimbripetalum* Turcz.) based on its fimbriate petals (Schischkin, 1936; Wu and Ke, 1996). Consistently, our results also support a distinct subclade (A1) for *S. radians* (**Figure 7**). Given its unique petal morphology and the possession of 2–5 seeds and reticulate seed surface, we propose recognizing this fimbriate-petaled group as a distinct section. Previously, this group contained only *S. radians*, and the recently described *Stellaria multipartita* Bo Xu & Meng Li which also possesses fimbriate petals is likely closely related (Song et al., 2020).

Our results reveal that species with conspicuous petioles form a cohesive grouping comprising subclades A2 and A3 (clade II; **Figure 7**), which is similar to the “Petiolares” clade identified in the global study (Sharples and Tripp, 2019). Correspondingly, classical taxonomy also grouped the petiolate species into series *Petiolares* Fenzl (Schischkin, 1936; Wu and Ke, 1996). Species in clades A2 and A3 are characterized by their stems often pubescent in 1 row or densely glandular hairy and its ovate to ovate-lanceolate leaves with distinct petioles on the middle and lower parts of the stem, rendering them readily distinguishable from other species in the genus. Our results support their recognition them as a distinct clade. In unsampled *Stellaria* species, *S. nemorum* L. will be encompassed within this clade for its broader leaves, stems densely glandular hairy and distinct petioles. However, we note that petiolate species also appear in other clades (e.g., *S. vestita* Kurz, *S. chinensis* Regel, *S. abaensis*). Although *Stellaria abaensis* possesses conspicuous petioles and was originally placed in series *Petiolares* by its author (Xu and Ma, 2018), our results indicate it is nested within clade F, rather than among the petiolate species (A2 and A3). This suggests that reliance solely on petiole characters may be inadequate for defining subgeneric groups.

The third major clade III contains the greatest number of species, including 15 subclades. This large clade is also present in the global study as the “Larbreae” clade (Sharples and Tripp, 2019). Correspondingly, classical taxonomy also grouped most species with generally narrower, all sessile or subsessile leaves, and usually angled but rarely smooth stems, into the subsection *Larbraea* (St. Hil.) Fenzl (Schischkin, 1936; Wu and Ke, 1996). Our results also support this large clade (clade III) including most species with glabrous or densely hairy stems (but never pubescent in 1 row or glandular), and sessile or short-petioled leaves.

Clade B consists of four species: *S. soongorica* Roshev., *S. filicaulis* Makino, *S. longifolia* Muhl. ex Willd., and *S. discolor* Turcz. (**Figure 7**). In the global study, three of these species belong to the “Parviflorae” clade, whereas *S. soongorica* was associated with the “Brachypetalae”, “Parviflorae”, and “Pedunculares” clades (Sharples and Tripp, 2019). The systematic position of *S. soongorica* remains unresolved (Sharples, 2019). Classically, *S. filicaulis* and *S. longifolia* were placed in series *Gramineae* Roshev., *S. discolor* in series *Discolores* Schischk., and *S. soongorica* in series *Pedunculares* Schischk (Schischkin, 1936; Wu and Ke, 1996). Although *S. soongorica* differs from other species by its brown or nearly black capsules and solitary flowers, it shares the following morphological traits with the species in clade B: plants are entirely glabrous, with quadrangular stems, linear sessile leaves ciliate at the base, petals 1.5–2 times as long as the sepals, and capsules significantly exceeding the persistent calyx. Our results support their recognition as a distinct clade.

Clade C is divided into two subclades. *Stellaria alsine* Grimm forms a distinct subclade (**Figure 7**), consistent with its treatment as a separate clade (the “Uliginosae” clade) in global studies (Sharples and Tripp, 2019). Classically, *S. alsine* and other glabrous species with wavy leaf margins were placed in series *Uliginosae* Schischk (Schischkin, 1936; Wu and Ke, 1996). Due to its 1–5 stamens and solitary flowers borne in leaf axils, we support its status as a distinct subclade. In subclade C2, *Stellaria salicifolia* Tsui ex P. Ke and *S. palustris* Ehrh. ex Retz. cluster together. In contrast, the global study grouped *S. salicifolia* with *S. chinensis* Regel in the “Chinenses” clade (Sharples and Tripp, 2019). A clear morphological distinction exists between *S. chinensis* (diffuse or decumbent stems, villous petiole, ovate to broadly ovate leaves with short-petiolate) and *S. salicifolia* (erect or ascending stems, entirely glabrous, linear and sessile leaves). Previous study also suggested a closer relationship between *S. chinensis* and *S. media* (Greenberg and Donoghue, 2011). Classically, *S. salicifolia* and *S. palustris* belong to series *Gramineae* (Schischkin, 1936; Wu and Ke, 1996). Given that *S. salicifolia* and *S. palustris* are entirely glabrous, possess petals equal in length to the sepals, and bear linear, sessile leaves, indicating a close relationship, our results propose them as a distinct subclade. In unsampled *Stellaria* species, *S. graminea* L. will be encompassed within this clade for its sharing above traits.

Species in the clade D are almost pubescent (except *Stellaria nipponica* Ohwi) (**Figure 7**). A corresponding clade (the “Vestitae” clade) exists in the global study, albeit with different species composition (e.g., *S. infracta* Maxim., *S. yunnanensis* Franch., *S. souliei* F. N. Williams) (Sharples and Tripp, 2019). Classically, species in this clade were distributed across series *Petiolatae*, series *Sessilifoliae* Y. W. Tsui ex P. Ke, and series *Parviflorae* Schischk for their sessile or short-petioled leaves and small flowers (Schischkin, 1936; Wu and Ke, 1996). Our results propose species with the following traits be assigned to a distinct subclade: plants are usually erect (rarely procumbent) and often covered in stellate or pubescent hairs; leaves are broader, stellate-pubescent or pubescent on both surfaces, and ciliate. Notably, *S. nipponica* within this clade is entirely glabrous. In the global study, *S. nipponica* was placed in the “Foliaceo-bracteatae” clade (Sharples and Tripp, 2019). This discrepancy might be due to cytonuclear discordance, and the systematic position of *S. nipponica* remains uncertain. However, it shares following the similar traits with the species in the clade B: plants are entirely glabrous, with quadrangular stems, linear sessile leaves ciliate at the base, petals 1.5–2 times as long as the sepals. We here propose grouping *S. nipponica* with the species in the clade B.

*S. sikkimensis* and *S. patens* in clade E possess straight soft hairs, broad leaves and high-altitude habitats. A corresponding clade (the “Patentes” clade) is present in the global study but includes additional species with differing indumentum types (not exclusively straight-pubescent, e.g., densely pubescent, minutely pubescent), and the support for that clade was weak (Sharples and Tripp, 2019). Classically, these two species were placed in series *Sessilifoliae* for their sessile leaves (Schischkin, 1936; Wu and Ke, 1996). However, based on our detailed field surveys at the type locality of *S. sikkimensis*, it has distinctly short-petiolate leaves, which is inconsistent with the diagnostic character of series *Sessilifoliae* characteristic. Our results demonstrate that the two species form a distinct and highly supported subclade, consistent with their shared morphological traits of straight soft hairs and ovate-lanceolate leaves.

Clade F comprises numerous Chinese endemics species characterized by relatively broad leaves and ciliate leaf margins. Within this clade, *Stellaria omeiensis* C. Y. Wu & Tsui ex P. Ke was placed in the “Vestitae” clade in the global study. Classically, species in this clade were placed in series *Sessilifoliae* for their sessile leaves (Schischkin, 1936; Wu and Ke, 1996). *S. abaensis* within this clade is particularly noteworthy. Although it presents conspicuous petioles and was proposed in series *Petiolatae*, our results support it far from the Petiolate clade (**Figure 7**). Our results indicated species sharing the following morphological traits form a distinct clade: plants are usually erect; stems are either cylindrical and glabrous, or quadrangular and pubescent; leaves are ovate to ovate-lanceolate, glabrous on both surfaces with ciliate margins, and the midrib is glabrous or hairy; leaves are usually sessile (except for *S. abaensis*, which is petiolate); inflorescences are terminal; and petals are subequal to the sepals. *Stellaria reticulivena* Hayata L. and *Stellaria media* var. *micrantha* (Hayata) T. S. liu & S. S. Ying will be encompassed within this clade for sharing the above traits.

Clade G is divided into five subclades (**Figure 7**). Subclade G1 contains the recently described species *Stellaria longipedicellata* W.Q.Wang & Z.H.Ma, which is morphologically similar to *Stellaria decumbens* Edgew. but differs in having a glabrous stem and longer petals (Wang et al., 2024). Our results confirm its close relationship to *S. decumbens*. Subclade G2 contains cushion-forming species including *S. decumbens* and its varieties (**Figure 7**). Similarly, a corresponding clade (the “Decumbentes” clade) exists in the global study, although *Stellaria polyantha* (Edgew. & Hook.f.) M.T.Sharples & E.Tripp (*Stellaria decumbens* var. *polyantha* Edgew. & Hook.f.) was placed in the “Patentes” clade (Sharples and Tripp, 2019). Classically, *S. decumbens* and its varieties were assigned to section *Adenonema* (Bge.) Pax on account of their cushion-like habit (Schischkin, 1936; Wu and Ke, 1996). Furthermore, Ke Ping proposed establishing a new series *Pulvinatae* Y. W. Tsui et P. Ke including *S. decumbens* and its varieties (Ke, 1985). Consistently, our results also present *S. decumbens* and its varieties as a monophyletic clade (**Figure 7**). We therefore propose retaining series *Pulvinatae* including those species in subclade G1 and G2. In unsampled *Stellaria* species, *Stellaria congestiflora* Hara. will be encompassed within this clade for its pulvinate habit. Although *Stellaria arenarioides* Shi L. Chen, Rabeler & Turland was nested in G4, we propose it will be encompassed within this series for its pulvinate habit.

Species in subclades G3 and G4 were classically placed in series *Petiolares*, series *Sessilifoliae*, series *Parviflorae*, series *Gramineae*, series *Uliginosae*, and section *Adenonema* (Schischkin, 1936; Wu and Ke, 1996). The global study placed *S. lanata* in the “Decumbens” clade, and the minutely pubescent *S. sikkimensis* and *S. mainlingensis* in the “Patentes” clade (Sharples and Tripp, 2019). Except for *Stellaria graminea* var. *viridescens* Maxim., all species are characterized by the presence of minute pubescence, soft hairs, or lanate hairs, which supports their classification as a distinct group. Then we also placed *Stellaria lanipes* C. Y. Wu & H. Chuang in this clade owing to its morphological similarity to *S. lanata*. Species in subclade G5 possess relatively broad and short-petiolate leaves, including *S. vestita* Kurz and *S. chinensis* Regel. Classically, they were placed in series *Petiolatae* on the basis of their short petiolate and broad leaves (Schischkin, 1936; Wu and Ke, 1996). Although both species exhibit short petiolate, our results reveal a distant relationship with clades A2 and A3, which contain the most distinctly petiolate species. In the global study, these two species were placed in the “Vestitae” and “Chinensis” clades, respectively (Sharples and Tripp, 2019). This clade may encompass *S. nepalensis* Majumdar & Vartak and *S. gyirongensis* L. H. Zhou for their relatively broad and short-petiolate leaves.

Clade H is divided into four subclades (**Figure 7**). Classically, the H1 species (*Stellaria uda* F. N. Williams) was placed in series *Parviflorae* for its small flower, whereas the global study assigned it to the “Decumbens” clade (Schischkin, 1936; Wu and Ke, 1996; Sharples and Tripp, 2019). Given its diffuse stem, distinct petal, and two lines of hairs on the stem, our results support *S. uda* as a distinct clade (**Figure 7**). Subclade H2 contains a newly described species, *Stellaria pentastyla* W.Q. Wang, H.F. Xu & Z.H. Ma, characterized by five styles and purple-black fruits (**Figure 7**) (Wang et al., 2020). On the basis of its unique style number and fruit color, our results support its recognition as a distinct clade. Classically, Subclade H3 species were placed in series *Foliaceo-bracteatae* Schischk. and series *Uliginosae* (Schischkin, 1936; Wu and Ke, 1996). The global study assigned them to the “Brevipetala” clade, while *Stellaria crassifolia* Ehrh. was placed in the “Umbellata” clade (Sharples and Tripp, 2019). Considering the significant inflorescence differences from *Stellaria irrigua* Bunge, we support their placement in H3. This clade is defined by a group of species sharing these traits: usually dwarf plants with predominantly glabrous stems (rarely pubescent apically); ovate-lanceolate to oblong-lanceolate, glabrous, sessile leaves; typically solitary or few-flowered cymose inflorescences; and capsules exceeding the persistent calyx. Based on the shared characteristics mentioned above, we propose assigning *S. depressa* Schmid and *S. imbricata* Bunge to this clade. Subclade H4 comprised *S. alaschanica* Y. Z. Zhao, *S. irrigua*, *S. parviumbellata* Y. Z. Zhao, *S. alsine* var. *alpina* (Schur) Hand.-Mazz., and *S. brachypetala* Bunge (**Figure 7**). Classically, *S. irrigua* and *S. parviumbellata* were placed in series *Umbellatae* Schischk., *S. alaschanica* in series *Parviflorae* (Schischkin, 1936; Wu and Ke, 1996). Similarly, a corresponding clade (the “Umbellatae” clade) also exists in the global study, which is largely congruent with our results. However, our results do not include *S. crassifolia* in this clade. Additionally, palynological studies indicated pollen grains possessing a spheroidal or spheroidal-polyhedral shape, 12–14 pores with sunken aperture membranes, and ornamentation of the microechinate-perforate, microechinate-punctate, or microechinate-punctate-perforate type with dense and relatively long microechinae are classified as the “*Stellaria umbellata* type” pollen, including *S. alsine* var. *alpina*, *S. irrigua*. Our results support the recognition of a clade characterized by the following traits: stems are glabrous or densely pubescent; leaves are ovate, linear-lanceolate, or ovate-lanceolate, glabrous on both surfaces and ciliate at the basal margins; inflorescences are corymbose cymes or few-flowered cymes; petals are absent or minute, typically shorter than the sepals by one-third (rarely slightly shorter); and capsules are 1.5–2 times as long as the sepals.

### 4.4 Two enigmatic “False *Stellaria*” species

In the most recent global study of *Stellaria*, the term “false *Stellaria*” has been proposed to refer to species whose petals are not deeply cleft and seeds number are less (Sharples, 2019; Sharples and Tripp, 2019). Our detailed investigation has identified several such cryptic “false *Stellaria*” species among the Chinese taxa.

The first is *Stellaria tibetica* Kurz. It possesses petals shallowly cleft to the middle and five stamens, characteristics that clearly exclude it from the genus *Stellaria* as currently circumscribed. Moreover, molecular studies indicate that its phylogenetic position is distant from the core *Stellaria* lineage (Arabi et al., 2022). Therefore, this taxon strongly warrants exclusion from *Stellaria* based on morphological evidence, pending future genomic validation. However, due to insufficient sampling, its precise systematic placement remains uncertain. Regrettably, our study also did not obtain material of this species, and further investigation is warranted.

The second case concerns *Stellaria henryi* F. N. Williams. Upon examination of the type specimen and original description, we determined that this species possess shallowly cleft petals, solitary flowers in the leaf axils, calyces and stems bearing purplish-black hairs, and five stamens — features that are clearly inconsistent with the current circumscription of *Stellaria*. Interestingly, within the global phylogenetic framework of *Stellaria*, *S. henryi* is deeply embedded within the genus, specifically in the Chinese clade (Sharples and Tripp, 2019). However, our examination of a specimen cited purportedly as this species revealed a cymose inflorescence, deeply cleft petals, and a completely glabrous stem, indicating that the specimen is misidentified. Under the current circumscription of *Stellaria*, species should have petals deeply 2-cleft to the base and typically ten stamens. Therefore, we strongly recommend the exclusion of this taxon from *Stellaria* based on morphology. This study also did not collect this particular species, pending future genomic validation.

Finally, *Stellaria yunnanensis* was previously suggested for exclusion from the genus based on its thick roots within global *Stellaria* studies (Sharples and Tripp, 2019). However, our research demonstrates that this species has petals deeply 2-cleft to the base and possesses cymose inflorescences and numerous seeds, clearly aligning with the diagnostic characteristics of *Stellaria*. Furthermore, other species within *Stellaria*, such as *S. pilosoides* Shi L. Chen, Rabeler & Turland, also possess stout roots. Our results indicate that *S. yunnanensis* is deeply nested in *Stellaria*. Similarly, the tribe Alsineae study also supported its position within *Stellaria* (Arabi et al., 2022). Additionally, palynological studies indicated that the pollen grains of *S. yunnanensis* are the “*Stellaria vestita* type”, characterized by a spheroidal shape and microechinate-perforate ornamentation, resembling the pollen morphology of other *Stellaria* species (Xu et al., 2020). Therefore, *S. yunnanensis* should remain accepted in *Stellaria*.

### 4.5 The Decumbentes clade and sect. *Plettkea*

The Decumbentes clade and sect. *Plettkea* are morphologically similar, both being cushion-forming and exhibiting comparable variation in petals. A key distinction is that *Stellaria decumbens* typically has ten stamens, whereas species of the new section mostly have five stamens. Notably, *S. decumbens* var. *arenarioides* L. H. Zhou within the Decumbentes clade also has five stamens and lacks petals, indicating a closely affinity to sect. *Plettkea*. Meanwhile, the sect. *Plettkea* forms a sister clade to either the Niten clade or the Larbreae clade, which includes *S. decumbens* (Montesinos-Tubée and Borsch, 2023). Unfortunately, that study did not include samples of *S. decumbens*. Our results recover the Decumbentes clade (G2 clade; **Figure 7**) as a distinct lineage, with cushion-forming species clustering together, and *S. decumbens* var. *arenarioides* also nested within this clade. It is possible that the proposed sect. *Plettkea* is closely related to the Decumbentes clade. Alternatively, both the new section and the Decumbentes clade occur at high altitudes, and their morphological similarity may result from convergent evolution. There appears to be a general trend toward convergent evolution within the genus *Stellaria*.

## 5 Conclusion

This study provides a thorough comparative analysis of the plastid genomes of *Stellaria*, revealing the relatively conserved structure of *Stellaria* plastid genomes and three promising DNA barcodes for *Stellaria*. Phylogenomic analysis provides new insights into the infrageneric relationships of *Stellaria* based on plastid genomes and morphology. Some clades are different from the recent study of *Stellaria*. This indicates that future taxonomic revisions should be based on phylogenies derived from multiple data sources, as well as the integration of multiple lines of evidence.

## Data Availability

The datasets presented in this study can be found in online repositories. The names of the repository/repositories and accession number(s) can be found in the article/**Supplementary Material**.

## Supplementary material

Supplementary Figure 1 Bayesian phylogenetic tree of *Stellaria* based on concatenated DNA barcoding.

Supplementary Figure 2 Relative synonymous codon usage values of the plastid genome of 61 *Stellaria* species.

Supplementary Table 1 Taxa sampled and the vouchers in infrageneric phylogenetic analysis of *Stellaria*.

Supplementary Table 2 Morphological features of *Stellaria* species.

Supplementary Table 3 Number of codons and relative synonymous codon usage values of the plastid genome of 61 *Stellaria* species.

Supplementary Table 4 Number of simple repeat sequences SSRs in 61 *Stellaria* plastid genomes.

Supplementary Table 5 Number of long repeats sequences in 61 *Stellaria* plastid genomes.
